# Transcriptome Sequencing and Identification of Cold Tolerance Genes in Hardy *Corylus* Species (*C. heterophylla* Fisch) Floral Buds

**DOI:** 10.1371/journal.pone.0108604

**Published:** 2014-09-30

**Authors:** Xin Chen, Jin Zhang, Qingzhong Liu, Wei Guo, Tiantian Zhao, Qinghua Ma, Guixi Wang

**Affiliations:** 1 Shandong Institute of Pomology, Shandong Provincial Key Laboratory of Fruit Tree Biotechnology Breeding, Tai'an, Shandong, China; 2 State Key Laboratory of Tree Genetics and Breeding, Research Institute of Forestry, Chinese Academy of Forestry, Beijing, China; 3 School of Forest Resources and Conservation, University of Florida, Gainesville, Florida, United States of America; Institute of Hydrobiology, Chinese Academy of Sciences, China

## Abstract

**Background:**

The genus *Corylus* is an important woody species in Northeast China. Its products, hazelnuts, constitute one of the most important raw materials for the pastry and chocolate industry. However, limited genetic research has focused on *Corylus* because of the lack of genomic resources. The advent of high-throughput sequencing technologies provides a turning point for *Corylus* research. In the present study, we performed *de novo* transcriptome sequencing for the first time to produce a comprehensive database for the *Corylus heterophylla* Fisch floral buds.

**Results:**

The *C. heterophylla* Fisch floral buds transcriptome was sequenced using the Illumina paired-end sequencing technology. We produced 28,930,890 raw reads and assembled them into 82,684 contigs. A total of 40,941 unigenes were identified, among which 30,549 were annotated in the NCBI Non-redundant (Nr) protein database and 18,581 were annotated in the Swiss-Prot database. Of these annotated unigenes, 25,311 and 10,514 unigenes were assigned to gene ontology (GO) categories and clusters of orthologous groups (COG), respectively. We could map 17,207 unigenes onto 128 pathways using the Kyoto Encyclopedia of Genes and Genomes Pathway (KEGG) database. Additionally, based on the transcriptome, we constructed a candidate cold tolerance gene set of *C. heterophylla* Fisch floral buds. The expression patterns of selected genes during four stages of cold acclimation suggested that these genes might be involved in different cold responsive stages in *C. heterophylla* Fisch floral buds.

**Conclusion:**

The transcriptome of *C. heterophylla* Fisch floral buds was deep sequenced, *de novo* assembled, and annotated, providing abundant data to better understand the *C. heterophylla* Fisch floral buds transcriptome. Candidate genes potentially involved in cold tolerance were identified, providing a material basis for future molecular mechanism analysis of *C. heterophylla* Fisch floral buds tolerant to cold stress.

## Introduction

The genus *Corylus* consists of deciduous species which naturally occur in temperate forest areas in Europe, the Middle East, Asia, and North America [1]. Currently over 4,000,000 t of nuts are commercially produced through the world, of which 700,000 t are hazelnut production [2]. Hazelnuts are important woody species in Northeast China. Its cultural area is approximately one million hectares, ranking first in the world. As the most widely distribution *Corylus* plant in China, *Corylus heterophylla* Fisch yield accounts for more than 70% of the total output in the domestic market of China [3]. Hazelnuts, due to their organoleptic characteristics, constitute one of the most important raw materials for pastry and chocolate industry [2], [4]. In addition to providing a desirable flavor to different food, they play an important role in human nutrition and health due to their protein, oil, vitamin, and mineral content. Hazelnuts are rich in both monounsaturated and polyunsaturated fatty acids, as well as in vitamin E [5]. *Corylus* species are also important sources of taxol (paclitaxel), which is an effective yet relatively expensive medicine for treatment of breast, ovarian, and lung cancer [6]–[8].

Cold stress is one of the most severe abiotic stresses and adversely affects plants by causing tissue injury and delayed growth [9], [10]. Cold stress can be classified as chilling (<20°C) and freezing (<0°C) stress [11]. Not all plants are always ready to tolerate freezing temperatures. However, many plants are tolerant of freezing temperature after exposure to non-freezing low temperature, a phenomenon called cold acclimation (CA) [12], [13]. *Arabidopsis* cold acclimate only about 5–7°C allowing for brief exposures to freezing temperatures whereas woody perennials can withstand extremely low subzero temperatures for extended periods of time. In addition, overwintering floral buds display both enhanced freezing tolerance and dormancy/relief of dormancy [14]. In such process, various physiological and biochemical changes occur in plant cells, which may confer subsequent acquired chilling and freezing tolerance to plants [15]. The signaling pathways used by plants in responding to cold stress and the key genes for modifying the response are of interest [16]. The best characterized regulon of cold-stress responses in plants contains transcription factor C-repeat binding factor dehydration-responsive element-binding protein (CBF/DREB) and its cold-inducible target genes, known as COR (cold-regulated gene), KIN (cold-induced gene), RD (responsive gene to dehydration), or LTI (low-temperature-induced gene) [17]–[22]. A large number of studies demonstrate that gene expression changes occur in a wide range of plant species in cold response [15], but the precise hierarchical organization of the global network has not been defined.

Hazelnuts will grow in a wide range of soil types from acidic mountain soils to basic soils derived from limestone. The plants grow best in mild, humid climate without extremes of heat or cold. However, buds, leaves, catkins, and female flowers are tolerant of frosts down to −7°C [23]. However, little is known about its tolerance mechanisms. During the last decades, large amounts of transcriptomic and genomic sequences have been available in many model organisms. For *Corylus heterophylla*, only 90 nucleotide sequences have been deposited in GeneBank database (as of the May 25, 2014). Therefore, extensive genomic or transcriptomic sequences are badly needed for *Corylus heterophylla*, which can be used for new genes discovery, gene localization, and comparative genomics and so on.

Transcriptome is the complete collection of transcripts in a cell at a specific developmental stage, which provides valuable and comprehensive information on gene expression, gene regulation and amino acid content of proteins [24]. The development of sequencing technology has provided a novel method for the analyses of transcriptome [25]. In plants, RNA-seq has accelerated the investigation of the complexity of gene transcription patterns, functional analyses and gene regulation networks [26]. In the present work, an RNA-seq project for *C. heterophylla* Fisch was initiated. Four *C. heterophylla* Fisch floral buds samples, including floral buds in non-cold acclimation (NA), cold acclimation (CA), midwinter (MW), and deacclimation (DA) stages were sequenced using the high-throughput Illumina deep sequencing technique. In addition, we estimated the expression profiles of key genes involved in cold acclimation. The transcriptome sequencing from *C. heterophylla* Fisch may help improve future genetic and genomic studies on the molecular mechanisms behind the cold tolerance of the *C. heterophylla* Fisch floral buds.

## Results and Discussion

### mRNA-seq and *de novo* transcriptome assembly

To obtain a global overview of the *C. heterophylla* Fisch floral buds transcriptome, a cDNA library was generated from an equal mixture of RNA isolated from floral buds in the four stages during winter (including NA, CA, MW, and DA), and paired-end sequenced using the Illumina platform. After stringent quality assessment and data filtering, 25,221,054 of 75-bp reads (∼1.85 G) with 97.38% Q20 bases (those with a base quality greater than 20) were selected as high quality reads for further analysis. An overview of the sequencing is presented in Table 1. The high quality reads produced in this study have been deposited in the National Center for Biotechnology Information (NCBI) SRA database (accession number: SRX529300).

**Table 1:** Summary of Illumina transcriptome sequencing for *C. heterophylla* Fisch.

Sample	Total Raw Reads	Total Clean Reads	Total Clean Nucleotides (nt)	GC (%)	Q20 (%)	N (%)
*C. heterophylla* Fisch	28,930,890	25,221,054	1,853,747,469	47.77	97.38	0.01

**Figure 1:**
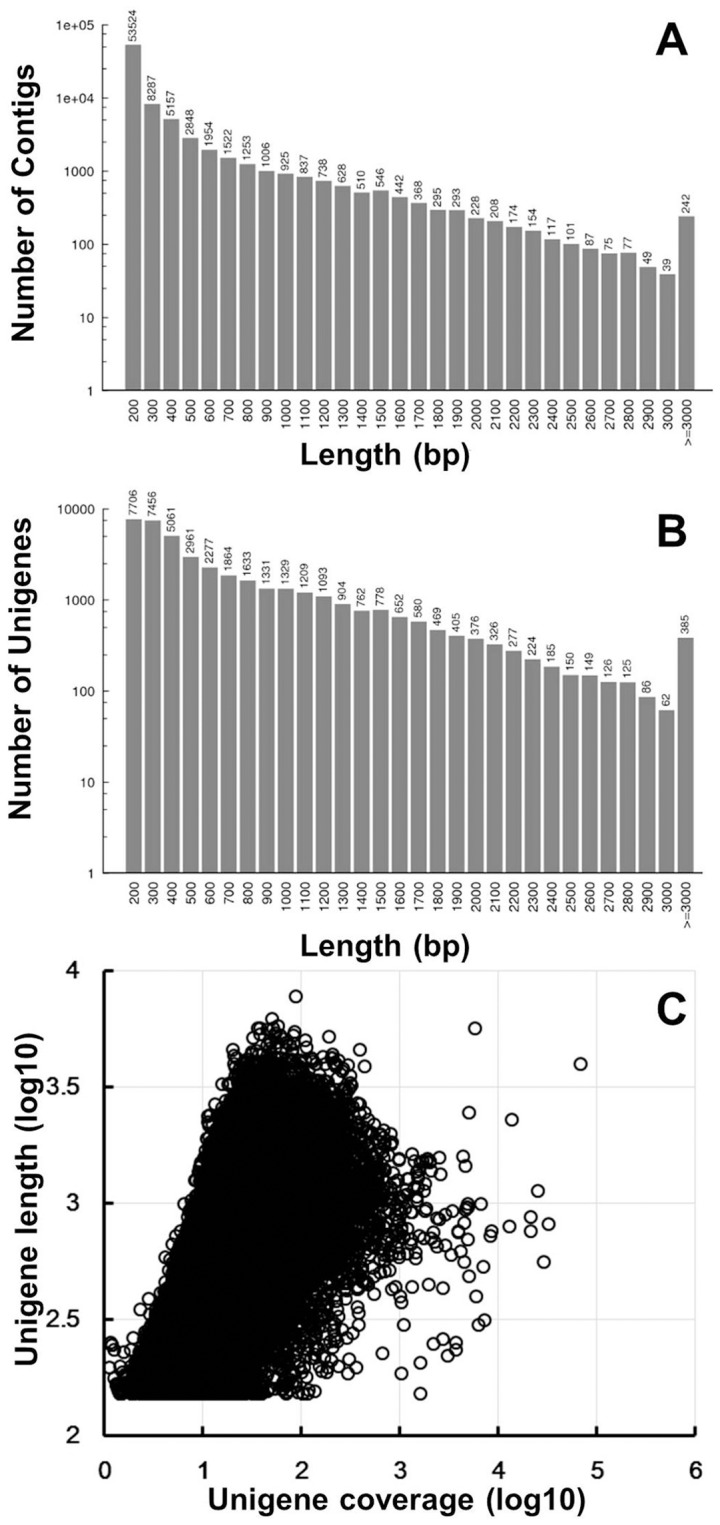
Overview of the *C. heterophylla* Fisch transcriptome sequencing and assembly. (**A**) Length distribution of *C. heterophylla* Fisch contigs.(**B**) Size distribution of *C. heterophylla* Fisch unigenes.(**C**) Log-Log plot showing the dependence of unigene length on the number of reads assembled into each unigene.

Using the Trinity *de novo* assembly program [27], next-generation short read sequences were assembled into 82,684 contigs, with N50 length of 642 bp and with mean length of 325 bp (Table 2). The distribution of contigs is shown in Fig. 1A. In total, there were 6,208 contigs coding for transcripts longer than 1 kb and 1,323 contigs coding for transcripts longer than 2 kb. The contigs were subjected to cluster and assembly analyses. A total of 40,941 unigenes were obtained, among which 9,323 genes (22.8%) were greater than 1 kb. The length distributions of unigenes are shown in Fig. 1B, revealing that more than 17,757 unigenes (43.4%) are greater than 500 bp. An overview of the assembled contigs and unigenes is presented in Table 2. These results demonstrated the effectiveness of Illumina pyrosequencing in rapidly capturing a large portion of the transcriptome. As expected for a randomly fragmented transcriptome, there was a positive relationship between the length of a given unigene and the number of reads assembled into it (Fig. 1C).

**Table 2:** Summary of Illumina transcriptome assembly for *C. heterophylla* Fisch.

Length	Total Length (Percentage)	
	Contigs	Unigenes
100–500	69,816 (84.44%)	23,184 (56.63%)
500–1000	6,660 (8.05%)	8,434 (20.60%)
1000–1500	3,259 (3.94%)	4,746 (11.59%)
1500–2000	1,626 (1.97%)	2,482 (6.06%)
≥2000	1,323 (1.60%)	2,095 (5.12%)
Total Length	26,907,332	27,778,721
Count	82,684	40,941
N50 Length	642	1128
Mean Length	325	679

### Transcriptome annotation

**Figure 2:**
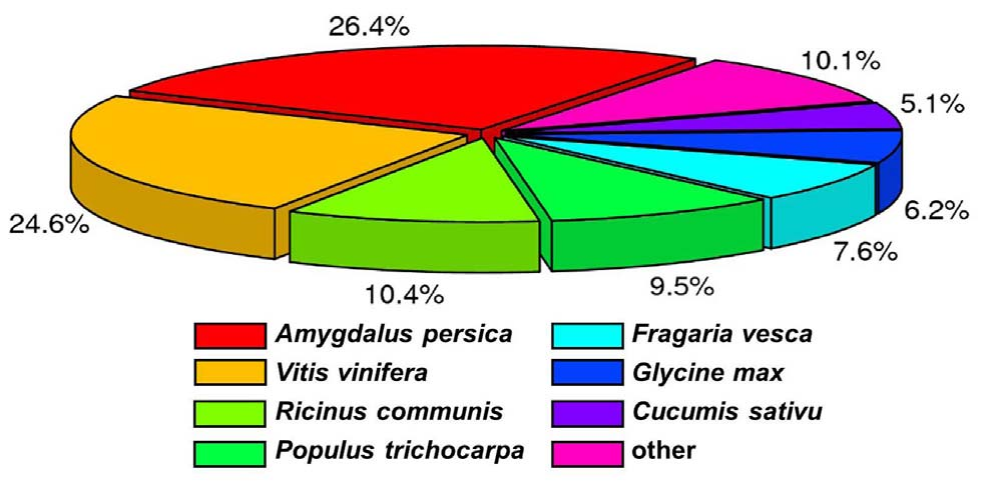
Comparative analysis of *C. heterophylla* Fisch unigenes with different species. Homology analysis of *C. heterophylla* Fisch unigenes with multiple species.

To determine protein-coding transcripts we screened the *C. heterophylla* Fisch floral buds transcriptome against the NCBI Non-redundant (Nr) peptide database and Swiss-Prot protein database using BLASTx with a cutoff *E*-value of 10^−5^. Mapping of 30,549 (74.6%) of the unigenes to the Nr library suggests that most of the unigenes can be translated into proteins. Furthermore, 18,581 (45.4%) unigenes had significant matches in the Swiss-Prot database (Table 3). Distribution analysis based on BLASTx searches showed that the unigenes of *C. heterophylla* Fisch have homologs in numerous hit a lot of plant species (Fig. 2). Among the various plant species that have protein sequence information in GenBank, the unigenes of *C. heterophylla* Fisch had the highest number of hits to sequences from *Amygdalus persica* (26.4%), followed by *Vitis vinifera* (24.6%), *Ricinus communis* (10.4%), *Populus trichocarpa* (9.5%), *Fragaria vesca* (7.6%), *Glycine max* (6.2%), and *Cucumis sativu* (5.1%) (Fig. 2). The high similarity of *C. heterophylla* Fisch unigenes to genes from *Amygdalus persica* and *Vitis vinifera* suggests the possibility of using the genome of *Amygdalus persica* or *Vitis vinifera* as a reference for identifying different gene expression patterns of mRNA-seq data.

**Table 3:** Functional annotation of the *C. heterophylla* Fisch transcriptome.

Annotated databases	All sequences	≥300 bp	≥500 bp	≥1000 bp	≥2000 bp
Nr Annotation	30,549	22,862	17,076	9,259	2,089
Swiss-Prot Annotation	18,581	14,707	11,477	6,752	1,668
COG Annotation	10,514	9,338	7,903	5,066	1,319
KEGG Annotation	17,207	13,808	10,885	6,587	1,646
GO Annotation	25,311	19,453	14,842	8,267	1,879
Total Annotation	31,844	22,889	17,080	9,260	2,089
All	40,941	25,779	17,757	9,323	2,095

### Functional classification by GO

**Figure 3:**
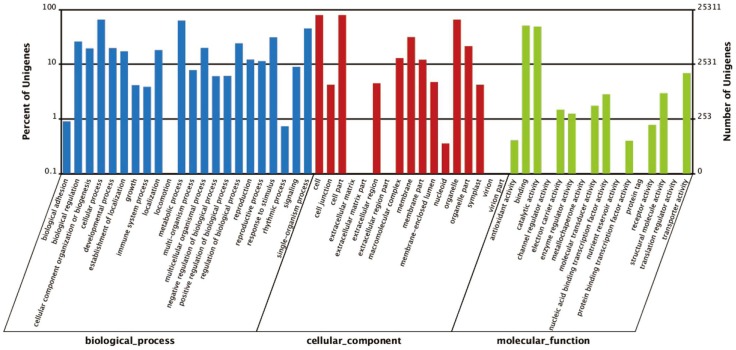
Functional annotation of assembled sequences based on gene ontology (GO) categorization. Results are summarized for three main Go categories: Biological Process, Molecular Function, and Cellular Component. Detail information of GO terms for all unigenes were listed in Table S2.

**Figure 4:**
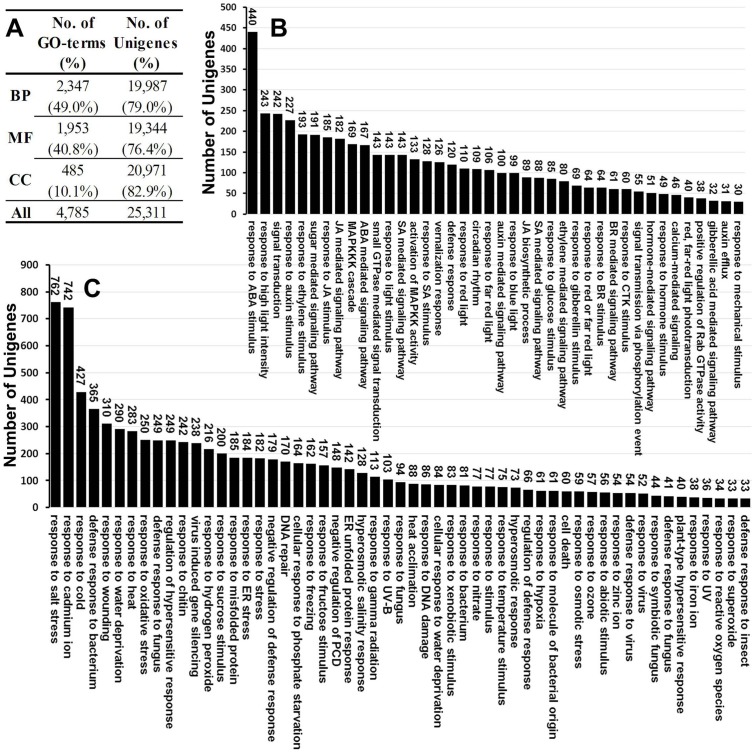
GO terms under response to stress and signal transduction. (**A**) Summarized of GO terms in Biological Process (BP), Molecular Function (MF), and Cellular Component (CC). (**B**) GO terms related to signal transduction. (**C**) GO terms related to stress response.

To assign functional information to transcripts, Gene Ontology (GO) analysis was carried out, which provides a dynamic, controlled vocabulary and hierarchical relationships for the representation of information on biological process, molecular function, and cellular component, allowing a coherent annotation of gene products. There were 30,549 unigenes annotated in Nr database, among which 25,311 unigenes were assigned with one or more GO terms, with 49.0% for biological process, 40.8% for molecular function, and 10.1% for cellular component (Fig. 3 and Fig. 4A). For biological process, metabolic process (GO:0008152) were the most represented GO term, followed by cellular process (GO:0009987). Regarding molecular function, unigenes with binding activity (GO:0005488) and catalytic activity (GO:0003824) were highly represented. For cellular components, the most represented category was cell (GO:0005623) and cell part (GO:004464) (Fig. 3).

Hardy plants develop essential tolerance for cold survival through multiple levels of biochemical and cell biological changes. These responses are due to reprogramming of gene expression which results in the adjusted metabolic alternations. The first step in switching on such molecular responses is to perceive the stress as it occurs and to relay information about it through a signal transduction pathway [28]. To explorer the unigenes might be involved in signal transduction and stress responses, we then compared the GO terms related with signal transduction (Fig. 4B) and stress responses (Fig. 4C). The top 5 represented signal related GO terms included response to ABA stimulus (GO:0009737, 440 unigenes), response to high light intensity (GO:0009644, 243 unigenes), signal transduction (GO:0007165, 242 unigenes), response to auxin stimulus (GO:0009733, 227 unigenes), and response to ethylene stimulus (GO:0009723, 193 unigenes) (Fig. 4B). While the top 5 represented stress related GO terms included response to salt stress (GO:0009651, 762 unigenes), response to cadmium ion (GO:0046686, 742 unigenes), response to cold (GO:0009409, 427 unigenes), defense response to bacterium (GO:0042742, 365 unigenes), and response to wounding (GO:0009611, 310 unigenes) (Fig. 4C).

### Functional classification by COG and KEGG

**Figure 5:**
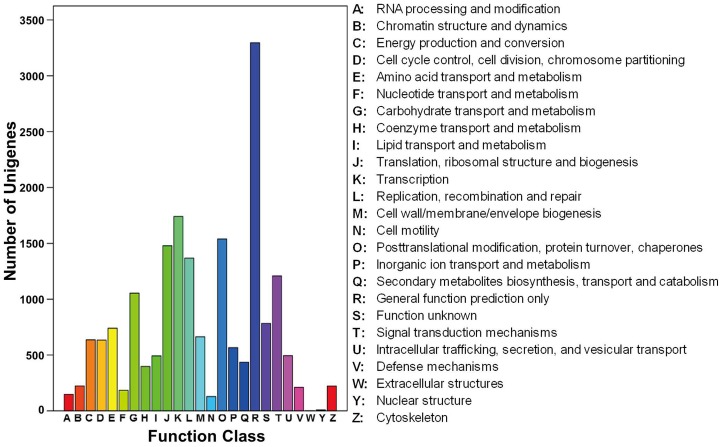
Clusters of orthologous groups (COG) classification. In total, 10,514 sequences were grouped into 25 COG classifications.

In addition, all unigenes were subjected to a search against the COG database for functional prediction and classification. Overall, 10,514 of the 40,941 sequences showing a hit with the Nr database could be assigned to COG classifications (Fig. 5). COG annotated putative proteins were functionally classified into at least 25 protein families involved in cellular structure, biochemistry metabolism, molecular processing, signal transduction and so on (Fig. 5). The cluster for general function prediction (3,296; 31.35%) represented the largest group, followed by transcription (1,741; 16.56%), posttranslational modification, protein turnover, chaperones (1,540; 14.65%), translation, ribosomal structure and biogenesis (1,478; 14.06%), replication, recombination and repair (1,367; 13.00%), signal transduction mechanisms (1,208; 11.49%), carbohydrate transport and metabolism (1,054; 10.02%), amino acid transport and metabolism (740; 7.04%), cell wall/membrane/envelope biogenesis (663; 6.31%), energy production and conversion (636; 6.05%), cell cycle control, cell division, chromosome partitioning (634; 6.03%), and whereas only a few unigenes were assigned to nuclear structure and extracellular structure (7 and 1 unigenes, respectively). In addition, 565 unigenes were assigned to inorganic ion transport and metabolism and 494 unigenes were assigned to intracellular trafficking, secretion and vesicular transport (Fig. 5).

**Figure 6:**
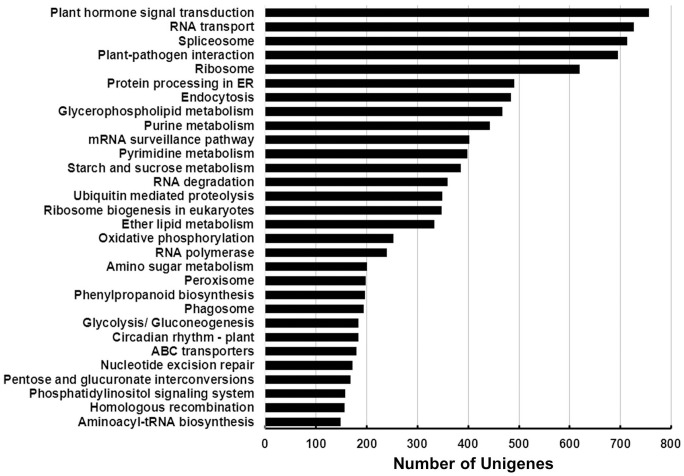
Distribution of *C. heterophylla* Fisch unigenes among Kyoto Encyclopedia of Genes and Genomes (KEGG) pathways. The top 30 most highly represented pathways are shown. Analysis was performed using Blast2GO and the KEGG database.

**Figure 7:**
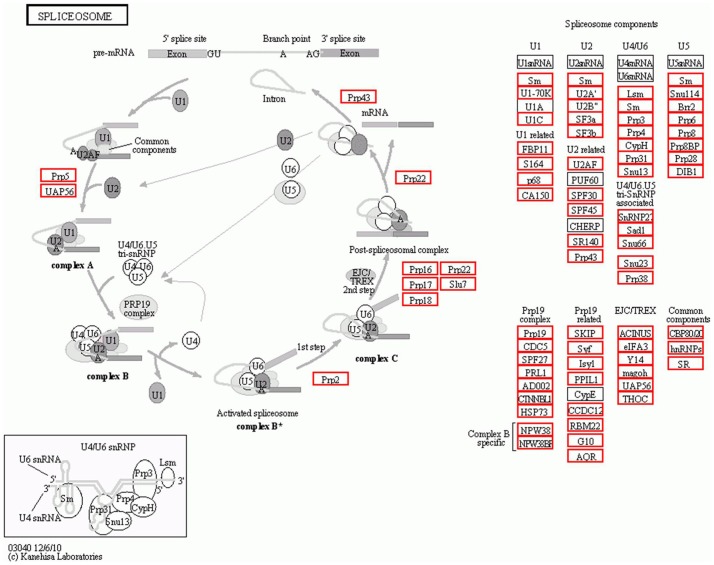
*C. heterophylla* Fisch unigenes (red rectangle) involved in spliceosome assembly pathway. Some *C. heterophylla* Fisch unigenes, such as Prp5, Prp2, Prp16, Prp12, and Prp43 etc, directly participated in the process of spliceosome assembly, while others made up the key components in spliceosome assembly including U1, U2, U4, U5, and U6 etc.

To further demonstrate the usefulness of *C. heterophylla* Fisch unigenes generated in the present study, we identified biochemical pathways represented by the unigene collection. Annotations of *C. heterophylla* Fisch unigenes were fed into the KEGG Pathway Tools, which is an alternative approach to categorize gene functions with the emphasis on biochemical pathways. This process predicted a total of 128 pathways represented by a total of 17,207 unigenes. Summary of the sequences involved in these pathways was included in Table S3. The top 3 pathways included Plant hormone signal transduction (756 unigenes), RNA transport (727 unigenes), and spliceosome (713 unigenes) (Fig. 6). As shown in Fig. 7, multiple *C. heterophylla* Fisch unigenes (red rectangle) were involved in the process of spliceosome assembly. Some made up the key components of spliceosome assembly including U1, U2, U4, U5 and U6 etc. Some other unigenes, such as Prp5, UAP56, Prp2, Prp16, Prp17, Prp18, Prp22, Slu7, and Prp43, directly participated in the process of spliceosome assembly. As *C. heterophylla* Fisch floral buds undergoing cold acclimation during winter and preparing for flower organs differentiation, this result showed that versatile alternative splicing events may occur in the *C. heterophylla* Fisch floral buds, which suggested that alternative splicing regulation is a general approach to affect complex plant biological processes including plant development, disease resistance and stress responses etc. Altogether, 31,844 (77.8%) unigenes were successfully annotated in the Nr, Swiss-Prot, COG, KEGG, and GO databases listed in Table S1.

### Candidate genes involved in cold tolerance in *C. heterophylla* Fisch floral buds

As we known, the response of plants to any environmental signal is mediated by a series of reactions, collectively referred to as signal transduction [28]. After perception of the cold signal, the downstream transcription factors and response genes were reprogrammed and then result in the adjusted metabolism. To identify the genes likely involved in cold tolerance in *C. heterophylla* Fisch floral buds, we construct a candidate cold tolerance gene set based on the GO terms representation. A large number of studies indicated that the homologs of selected candidate genes involved in plant cold tolerance (Table 4). The selected unigene ID and annotation are listed in Table 4.

**Table 4:** Candidate genes involved in cold tolerance in *C. heterophylla* Fisch floral buds.

Unigene ID	BLASTn Hit	*E-value*	Gene Name	RPKM	References
Unigene24785	Beta-amylase [*Prunus persica*]	0.00E+00	**BAM**	42.38	[43]
CL4405.Contig2	Potassium channel AKT1-like [*Cucumis sativus*]	0.00E+00	KT	2.00	[44]
CL3300.Contig1	Asparagine synthetase 1 [*Vitis vinifera*]	0.00E+00	ASNS	33.28	[45]
Unigene23215	Betaine-aldehyde dehydrogenase [*Corylus heterophylla*]	0.00E+00	BADH	53.50	[46]
CL1670.Contig2	Sucrose-phosphate synthase [*Citrus unshiu*]	0.00E+00	SPS	181.59	[47]
Unigene11928	Delta 1-pyrroline-5-carboxylate synthetase [*Gossypium arboreum*]	0.00E+00	P5CS	26.10	[48]
CL28.Contig4	Sensitive to freezing [*Ricinus communis*]	0.00E+00	SFR6	0.57	[49]
Unigene26540	CBL-interacting serine/threonine-protein kinase 5-like [*Cucumis sativus*]	0.00E+00	CIPK	30.94	[50]
CL1657.Contig1	Mitochondrial alternative oxidase 1A [*Corylus heterophylla*]	0.00E+00	AOX1A	1.11	[51]
CL371.Contig1	Enolase [*Corylus heterophylla*]	0.00E+00	ENO	49.23	[52]
CL2001.Contig2	MAP kinase [*Populus trichocarpa*]	0.00E+00	MAPK	2.07	[53]
Unigene24663	Omega-6 fatty acid desaturase, endoplasmic reticulum isozyme 2-like [*Fragaria vesca*]	0.00E+00	FAD2	368.94	[54]
CL569.Contig5	Calcium-dependent protein kinase 4-like [*Cucumis sativus*]	0.00E+00	CDPK	1.70	[55]
CL3389.Contig1	Phosphoinositide phospholipase C 2 isoform 2 [*Vitis vinifera*]	4.00E−179	PLC	11.89	[56]
CL1380.Contig2	Cold-induced protein [*Vitis vinifera*]	1.00E−169	**CIP**	5.53	[57]
Unigene12569	Spermidine synthase 1-like [*Fragaria vesca*]	1.00E−169	SPDS	61.34	[58]
CL3248.Contig2	Protein phosphatase 2C [*Prunus persica*]	2.00E−165	PP2C	7.57	[59]
CL539.Contig1	Phospholipase d beta, putative [*Ricinus communis*]	1.00E−156	PLD	15.21	[56]
Unigene515	Glycerol-3-phosphate acyltransferase [*Citrus unshiu*]	5.00E−150	**GPAT**	23.50	[60]
CL382.Contig1	Class IV chitinase [*Corylus heterophylla*]	3.00E−142	CHI	22.23	[61]
Unigene3155	Nitrate Reductase (NADH) [*Ricinus communis*]	8.00E−125	**NIR**	6.51	[62]
Unigene23517	Superoxide dismutase [Mn] [*Vitis vinifera*]	1.00E−114	Mn-SOD	110.73	[63]
CL3124.Contig4	Galactinol synthase 3 [*Populus trichocarpa*]	1.00E−109	GOLS	3.77	[64]
Unigene23440	Heat shock protein 22 [*Corylus heterophylla*]	3.00E−103	**Hsp22**	22.52	[65]
Unigene117	14-3-3 [*Prunus persica*]	1.00E−102	14-3-3	10.62	[66]
CL3168.Contig1	CBF/DREB1 transcription factor 1 [*Betula pendula*]	6.00E−94	CBF1	77.68	[11]
Unigene21005	Early-responsive to dehydration protein [*Populus trichocarpa*]	6.00E−92	**ERD7**	11.82	[33], [67]
CL100.Contig2	NAC family protein [*Corylus heterophylla*]	4.00E−82	NAC	19.627	[68]
Unigene18268	COR414-TM [*Vitis vinifera*]	2.00E−71	COR414	160.5406	[31], [69]
CL2732.Contig3	Transcription factor hy5, putative [*Ricinus communis*]	1.00E−69	HY	11.5457	[70]
CL1909.Contig1	Fatty acid desaturase 3 [*Corylus heterophylla*]	6.00E−61	FAD3	7.6633	[71]
CL2284.Contig2	Homeobox protein, putative [*Ricinus communis*]	4.00E−54	**HD-ZIP**	2.516	[72]
Unigene22531	bZIP transcription factor [*Vitis vinifera*]	1.00E−51	**bZIP78**	17.6696	[32]
Unigene6581	MYB transcription factor [*Populus trichocarpa*]	1.00E−46	MYB	9.0585	[20]
Unigene21495	AP2/ERF domain-containing transcription factor [*Populus trichocarpa*]	1.00E−41	ERF	48.4576	[73]
Unigene1447	Choline monooxygenase [*Camellia sinensis*]	1.00E−40	CMO	6.152	[74]
CL1909.Contig2	Omega-3 fatty acid desaturase [*Betula pendula*]	1.00E−35	FAD8	7.5632	[75]
Unigene5353	Beta-ketoacyl-ACP synthase [*Prunus persica*]	3.00E−33	KAS	10.0582	[76]
Unigene10730	ICE-like [*Cucumis sativus*]	1.00E−31	ICE	7.3351	[77]
Unigene12179	Actin depolymerizing factor [*Prunus persica*]	3.00E−14	**ADF**	57.5651	[35], [78]
CL4024.Contig1	Lipid transfer protein precursor [*Gossypium hirsutum*]	4.00E−14	LTP	11.4457	[79]
Unigene5522	COR413-PM1 [*Arabidopsis thaliana*]	4.00E−11	**COR413**	9.2351	[31], [80]

**Figure 8:**
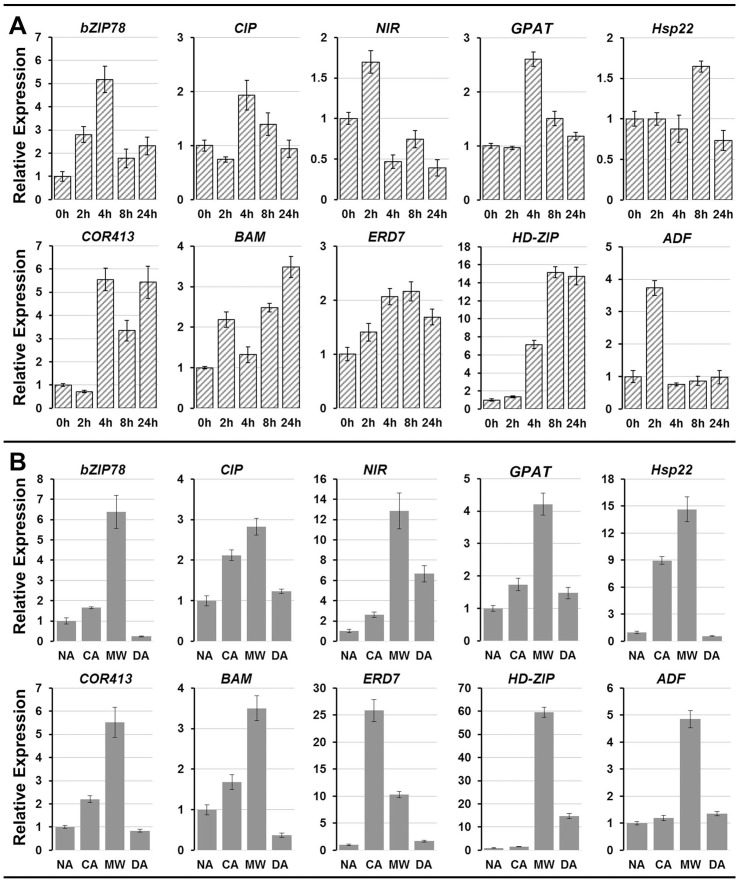
qRT-PCR analysis of 10 cold tolerance candidate unigenes in *C. heterophylla* Fisch. (**A**) Time course expression pattern of 10 cold tolerance candidate unigenes under 4°C cold stress (0 h, 2 h, 4 h, 8 h, 24 h) in *C. heterophylla* Fisch leaves. (**B**) Expression pattern of 10 cold tolerance candidate unigenes during four stages (NA, CA, MW, and DA) of overwintering in *C. heterophylla* Fisch floral buds. The gene names and the primers used for qRT-PCR analysis are shown in Table S4. Standard error of the mean for three biological replicates (nested with three technical replicates) is represented by the error bars.

To validate the responsible of genes in the candidate cold tolerance gene set to cold, we then selected ten genes from the set and detected their expression pattern under cold stress and during winter by qRT-PCR. In woody perennials of the temperate zone, cold acclimation is triggered by several environmental cues, not only low temperatures, and is generally considered a two-step process: The first stage is induced by short photoperiod and the timing and speed of acclimation can be affected by other such as available moisture. The second stage is induced by low temperature [29]. To exclude the influence of other factors, we first detected the time course expression patterns of these genes under cold stress (4°C). As shown in Fig. 8A, in *C. heterophylla* Fisch leaves, *NIR* and *ADF* were rapidly induced at 2h after treated by cold stress, while *bZIP78*, *CIP*, *GPAT*, and *COR413* were dramatically induced at 4h after treated by cold stress. On the whole, all the ten cold tolerance candidate genes were induced in different degree in *C. heterophylla* Fisch leaves when they were exposed to the cold stress. It suggests that these genes are responsible to cold stress. We then analyzed their expression pattern during overwintering in *C. heterophylla* Fisch floral buds. During winter, the *C. heterophylla* Fisch floral buds undergoing four stages: NA, CA, MW, and DA (see methods). These selected genes can be classified into three types according their expression patterns during the process of cold acclimation (Fig. 8B). (1) Type I: the expression of *bZIP78*, *CIP*, *NIR*, *GPAT*, *Hsp22*, *COR413*, and *BAM* were induced immediately in CA stage and were further induced in the following MW stage, indicating that these genes could response to early and later cold acclimation. (2) Type II: *ERD7* had higher expression in CA stage than MW stage, indicating *ERD7* might be mainly involved in early cold acclimation. (3) Type III: *HD-ZIP* and *ADF* were significant induced in MW stage, but were not induced in CA stage, implying their involvement in later response to cold stress.

In type I class, the mRNA level of membrane protein (COR413) and transcription factors (bZIP78) were immediately induced when the *C. heterophylla* Fisch floral buds into the CA stage and were further induced in MW stage. The cold-regulated (COR)413-plasma membrane and COR413-thylakoid membrane groups are potentially targeted to the plasma membrane and thylakoid membrane, respectively. It is known that the plasma membrane is the primary site of freezing injury [30]. As an integral membrane protein, COR413-PM could play a structural role by stabilizing the plasma membrane lipid bilayer [31]. Many studies have shown that bZIP transcription factor play an important role in the ability of plants to withstand various stresses. In soybean plants, *GmbZIP44*, *GmbZIP62*, and *GmbZIP78* can bind to GLM (GTGAGTCAT), ABRE (CCACGTGG), and PB-like (TGAAAA) elements with differential affinity. They may function in ABA signaling through upregulation of ABI1 and ABI2 and play roles in salt and freezing tolerance through regulation of various stress responsive genes [32]. The early response of these transcription factors could enhance the expression of a series downstream stress responsive genes rapidly and then enhance the cold tolerance of *C. heterophylla* Fisch floral buds. Type II class gene *ERD7* mainly induced in CA stage but not in MW stage. *Early responsive to dehydration* (*ERD*) genes are defined as genes that are rapidly activated during drought stress. ERD15 from *Arabidopsis* has been functionally characterized as a common regulator of the abscisic acid (ABA) response and the salicylic acid (SA)-dependent defense pathway [33]. The intense induction of *ERD7* in CA stage implying the hormone signal involved in early cold acclimation in *C. heterophylla* Fisch floral buds. In type III class, the actin depolymerizing factors (ADF) are part of the ADF/cofilin group, a family of small proteins (15–22 kD) that includes cofilin, destrin, depactin, and actophorin. The members of this family can be described as stimulus-responsive modulators of the cell actin cytoskeleton dynamics. Using *Arabidopsis* ADF1, Carlier *et al.* [34] have suggested that one of the main functions of ADF is to increase the turnover rate of actin filaments. In wheat, the induction of an active ADF during cold acclimation and the correlation with an increased freezing tolerance suggest that the protein may be required for the cytoskeletal rearrangements that may occur upon low temperature exposure [35]. Moreover, the kinetics of TaADF protein expression is different from the mRNA expression pattern [36]. During the acclimation period, *TaADF* mRNA level is maximal after 1 or 2 days and slowly decreases afterward, whereas protein accumulation increases and peaks at 49 days in the hardy cultivars. In this study, the expression of ADF was induced in MW stage in *C. heterophylla* Fisch floral buds, suggesting important changes in the actin cytoskeletal architecture may occur during later cold acclimation.

## Conclusion

In this study, *de novo* transcriptome sequencing of the *C. heterophylla* Fisch floral buds using Illumina platform was performed for the first time. 28,930,890 raw reads were *de novo* assembled into 40,941 unigenes. All unigenes were then evaluated and functionally annotated by comparing with the existing protein databases, such as NCBI Nr database, Swiss-Prot database, COG database, and KEGG database. A large number of candidate genes potentially involved in growth, development, and stress tolerance were identified, and are worthy of further investigation. To our knowledge, this is the first application of Illumina paired-end sequencing technology to investigate the transcriptome of *C. heterophylla* Fisch floral buds and moreover the assembly of the reads was conducted without reference genome information. The database will improve our understanding of the molecular mechanism of cold tolerance in *C. heterophylla* Fisch floral buds. This resource should lay an important foundation for future genetic or genomic studies on *Corylus* genus.

## Materials and Methods

### Plant materials and RNA extraction

*C. heterophylla* Fisch was obtained from Mulan Paddock, Hebei, China (116°32′–118°14′E, 4 1°35′–42°40′N). Plant samples collection was permitted by the State Forestry Administration and Forestry Bureau of Hebei province. For the field experiment, buds were collected starting in the fall of 2011 and through the winter of 2011–2012. Buds collected at the first time point in the fall (on Sep 29, 2011) were used as the NA control. They had received 0 chill units (number of hours between 0 and 7°C). For subsequent time points, buds were collected later in the fall when they had accumulated 198 chill units (CA stage, Nov 2, 2011), during midwinter when they had accumulated 682 chill units and had reached a maximum bud cold hardiness level of −29°C (MW stage, Dec 29, 2011), and during spring when they had accumulated 1,056 chill units and had partially deacclimated to a bud cold hardiness level of 15°C (DA stage, Apr 24, 2012). Floral buds were dissected from the hazelnut and immediately frozen and stored in liquid nitrogen until use.

For the short-term cold treatment, the seedlings were grown in a growth chamber with 25°C/21°C (day/night), 16 h light/8 h dark cycle. For the exposure to cold stress, four-weeks-old seedlings were transferred to a cold chamber at 4°C. The leaves were collected at 0, 2, 4, 8, and 24 h of cold stress. All of the experiments were repeated at least three times.

Total RNA was extracted from floral buds using the RNeasy Plant Mini kit (Qiagen, Valencia, CA, USA). DNA contamination was removed with RNase-free DNase I (Qiagen). RNA was concentrated and purified with an RNA MinElute kit (Qiagen). RNA quality and quantity were assessed by absorption at 260 nm/280 nm, gel electrophoresis, and via the Agilent 2100 Bioanalyzer (Agilent Technologies, USA).

### mRNA-seq library construction for illumine sequencing

The mRNA-seq library was constructed following the manufacturer's instructions of mRNA-seq Sample Preparation Kit (Cat# RS-930-1001, Illumina Inc, San Diego, CA) (Illumina). Briefly, mRNA was purified from 20 µg of total RNA using oligo (dT) magnetic beads. Following purification, the mRNA is fragmented into small pieces using divalent cations under elevated temperature. Taking these short fragments as templates, first-strand cDNA was synthesized using reverse transcriptase and random primers. Second-strand cDNA synthesis was followed using DNA polymerase I and RNase H. Sequencing adapters were ligated to short fragments after purifying with QiaQuick PCR extraction kit, which were used to distinguish different sequencing samples. Fragments with lengths ranging from 200 to 500 bp were then separated by agarose gel electrophoresis and selected for PCR amplification as sequencing templates. The final cDNA library was sequenced using Illumina GAIIx system according to the manufacturer's protocols, with a read leangth of pari-end (PE) 75 bp. The transcriptome datasets are available at the NCBI Sequence Read Archive (SRA) with the accession number SRX529300.

### Sequence data analysis and *de novo* assembly

The raw reads were cleaned by removing adaptor sequences, empty reads and low quality sequences, which included the reads with N percentage (i.e., the percentage of nucleotides in read which could not be sequenced) over 10% and ones containing more than 50% nucleotides in read with Q-value≤5. Transcriptome *de novo* assembly was performed separately with the short reads assembling programs SOAPdenovo [37] and Trinity [27]. It has been demonstrated that Trinity is a more efficient *de novo* transcriptome assembler, especially in the absence of a reference genome [27]. First, Trinity combined the reads with a certain overlap length to form longer fragments, which were called contigs. Next, these reads were mapped back to contigs; with paired-end reads, Trinity was able to detect contigs from the same transcript and determine the distances between these contigs. Finally, Trinity connected these contigs into sequences that could not be extended on their end. Such sequences were defined as unigenes.

As the Trinity assembler discards low coverage *k*-mers, no quality trimming of the reads was performed prior to the assembly. Trinity was run on the paired-end sequences with the fixed *k*-mer size of 25, minimum contig length of 100.

### Gene annotation and classifications

The optimal assembly results were chosen according to the assembly evaluation. The assembled sequences were compared against the NCBI Nr database and Swiss-Prot database using BLASTn with an *E*-value of 10^−5^. Gene names were assigned to each assembled sequence based on the best BLAST hit (highest score). To increase computational speed, such search was limited to the first 10 significant hits for each query. The ORFs were identified as the nucleotide sequence or as the protein translation provided by the “GetORF” program from the EMBOSS software package [38]. The longest ORF was extracted for each unigene. We quantified transcript levels in Reads Per Kilobase of exon model per Million mapped reads (RPKM) [39]. The RPKM measure of read density reflects the molar concentration of a transcript in the starting sample by normalizing for RNA length and for the total read number in the measurement. Genes with high expression levels were screened and listed.

To annotate the assembled sequences with GO terms describing biological processes, molecular functions and cellular components, the Swiss-Prot BLAST results were imported into Blast2GO [40], a software package that retrieves GO terms, allowing gene functions to be determined and compared. These GO terms are assigned to query sequences, producing a broad overview of groups of genes catalogued in the transcriptome for each of three ontology vocabularies, biological processes (BP), molecular functions (MF) and cellular components (CC). The unigenes sequences were also aligned to the COG database to predict and classify functions. KEGG pathways were assigned to the assembled sequences using the online KEGG web server (http://www.genome.jp/kegg/) [41]. The output of KEGG analysis includes KO assignments and KEGG pathways that are populated with the KO assignments.

### Quantitative RT-PCR

Total RNA was isolated from *C. heterophylla* Fisch floral buds during four stages (NA, Non-cold Acclimation; CA, Cold Acclimation; MW, Midwinter; DA, Deacclimation) with the RNeasy Plant Mini Kit (Qiagen, Valencia, CA, USA). cDNA synthesis was performed with 1 µg total RNA using the Superscript III First Strand Synthesis system followed by the RNase H step (Invitrogen, Carlsbad, USA), according to the manufacturer's protocol. Primer pairs were designed using Primer3 (http://frodo.wi.mit.edu/primer3/) with the following parameters: Tm of approximately 60°C, product size range of 100–260 base pairs, primer sequences with a length of approximately 20 nucleotides, and a GC content of 45–55%. The gene names and primers used for qRT-PCR are liste in Table S4. To quantify the expression level the selected genes, the *C. heterophylla* Fisch Actin was used as an internal control. qRT-PCR was performed using a 7500 Real-time PCR System (Applied Biosystems, CA, USA) and a SYBR Premix Ex Taq Kit (TaKaRa, Dalian, China). The relative quantitative method (ΔΔC_T_) was used to calculate the fold change of the target genes [42]. Three biological replicates (nested with three technical replicates) per sample were carried out.

## Supporting Information

Table S1 **Sequences with significant BLASTn matches against Nr, Swiss-Prot, COG, KEGG, and GO database.**
(XLSX)

Table S2 **GO terms for all unigenes of** ***C. heterophylla*** **Fisch.**
(XLSX)

Table S3 **KEGG biochemical pathways of** ***C. heterophylla*** **Fisch.** In order to better understand the biological functions of *C. heterophylla* Fisch unigenes, a total of 17,207 unigenes were assigned to 128 KEGG biochemical pathways.
(XLSX)

Table S4 **The sequences of qRT-PCR primers.**
(XLSX)
